# Surgical Task Shifting Helps Reduce Neonatal Mortality in Ethiopia: A Retrospective Cohort Study

**DOI:** 10.1155/2019/5367068

**Published:** 2019-02-03

**Authors:** Yihun Tariku, Tadele Gerum, Mareshet Mekonen, Haddis Takele

**Affiliations:** ^1^Department of Public Health, Arba Minch College of Health Sciences, Arba Minch, Ethiopia; ^2^Department of Public Health, College of Medicine and Health Sciences, Wolkite University, Wolkite, Ethiopia; ^3^Department of Midwifery, College of Medicine and Health Sciences, Arba Minch University, Arba Minch, Ethiopia; ^4^Departement of Surgery, Arba Minch General Hospital, Arba Minch, Ethiopia

## Abstract

**Background:**

To improve access to surgical service and to reduce neonatal mortality Ethiopia implemented surgical task shifting to nonphysician surgeons (NPSs). We aim at assessing surgical outcomes between NPSs and physician surgeons working in two hospitals.

**Methods:**

We collected data from two hospitals on 474 maternal medical records. Completed maternal medical records were included in this study. Data were entered into Epi Info version 7 and analyzed by SPSS version 20 software. Both descriptive and inferential statistics were done. If the 95% confidence interval values exclude the null value, the factor was considered as a significant factor.

**Result:**

Totally, 3429 mothers delivered in two hospitals. Of them, 840 (24.5%) delivered by caesarian section (CS), but 474 mothers' records meet the inclusion criteria included in this study. Of 474 CS deliveries, the majority (82%) of them were performed by NPS. Maternal or fetal emergency conditions were the main reasons (92.0%, *n*=436) for CS. Task shifting does not affect immediate newborn outcomes (ARR, 1.24 (0.55, 2.78)), but duration of hospitalization (ARR: 4 (2.3, 7.5)), condition of the fetus during admission (ARR: 5.22 (2.9, 9.2)), and type of anesthesia used (ARR: 0.2 (0.1, 0.4)) significantly determine the outcome.

**Conclusion:**

Surgical task shifting to NPS does not affect the immediate newborn outcome. But general anesthesia is one of the major factors that affects the outcome.

## 1. Introduction

Surgical care task shifting to nonphysicians is a common practice in most developing countries [1]. Ethiopia implemented task shifting to NPSs, (they are BSc health officers or BSc nurses plus a 4-year training on integrated emergency surgery and obstetrics) to overcome the dire shortage of physicians, to improve access to medical and surgical care service, and to reduce maternal and neonatal mortality [2–6].

Neonatal mortality is one of the major health problems of Ethiopia. Surgical task shifting is an intervention supposed to reduce the rate of neonatal mortality [7–9]. Emergency Obstetric and Newborn Care (EmONC) is another strategy to reduce neonatal mortality [10]. EmONC is best implemented in developing countries through surgical task shifting. There is evidence from different countries on significant reduction of neonatal mortality due to EmONC implementation [7, 11]. This is because surgical intervention of EmONC creates a window to save the life of both mother and fetus due to emergency conditions during pregnancy. An emergency condition is a major reason for CS [12–18].

Different scholars reveal that there is an improvement on access to CS service and reduction of neonatal mortality after implementation of surgical task shifting [1, 12–14, 19]. There are findings from Ethiopia that assess the quality of CS service and its rate; but this research assesses the effect of surgical task shifting on neonatal birth outcomes [12, 15, 16, 20, 21].

## 2. Methods and Materials

A retrospective cohort study design was used to assess the rate of CS delivery and the relationship between NPS and CS outcomes. Data were collected from July 1 to 30, 2017. The study was conducted in Arba Minch General Hospital and Sawla District Hospital. These hospitals were selected because they qualify Comprehensive EmONC standard [20].

Mothers who gave birth by CS from July 2015 to June 2016 and all those who meet inclusion criteria (fully recorded medical record, mothers who give birth after arrival to the hospital, and both the mother and fetus are alive at the time of arrival to the hospitals) were included in this study. Data were collected by data abstraction tools adopted from averting maternal death and disability program module [10]. We collected data on the condition of mother and fetus during admission, who conducts CS, different tasks done during the procedure, and status of the newborn until the time of discharge. Experienced midwives and anesthetists were used to collect data after attending one-day training on how to collect the data. Ethical clearance was obtained from the South Region Health Research Ethics Review Committee.

Data were entered into Epi Info version 7 and analyzed using SPSS version 20. We did both descriptive and analytic statistics. Initially, binary logistic regression was performed to assess the association between each exposure and outcome variables. During bivariate analysis, variables with *P* value 0.2 were considered for multivariate binary logistic regression. When 95% confidence interval values exclude null value the factors considered as a significant factor. The immediate newborn outcome was categorized into good and bad. The good outcome means when the newborn is alive during birth without distress, and bad outcome means when there is a prenatal death or lives birth with distress.

## 3. Results and Discussion

### 3.1. Results

#### 3.1.1. Participants in the Study

**Figure 1:**
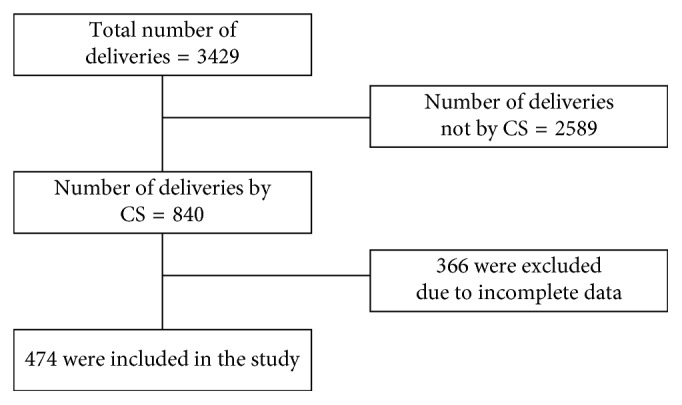
Schematic presentation of sampling procedure at selected health institutions, July 2015–June 2016 (*n*=474).

Totally, 3429 deliveries were attended in two hospitals, and of them 840 (24.5%) were delivered by CS. But only 474 mothers who fulfilled the inclusion criteria were included in this study (Figure 1).

#### 3.1.2. Characteristics of Mothers

From the total population, the majority (58%) of mothers were in the age group between 15 and 25: 189 (39.5%) mothers were nulliparous (not given birth before), and 64 (13.5%) mothers have more than 5 children. Greater than 60% of mothers were rural by residence, and 222 (48.2%) mothers come to hospital referred from rural health centers. At the time of admission, 159 (33.5%) mothers and 157 (33.1%) fetuses had an unstable vital sign. Only 152 (32.1%) mothers' medical registrations have data on the time interval from decision to perform CS to actual incision time.

#### 3.1.3. Indications for Cesarean Section

**Figure 2:**
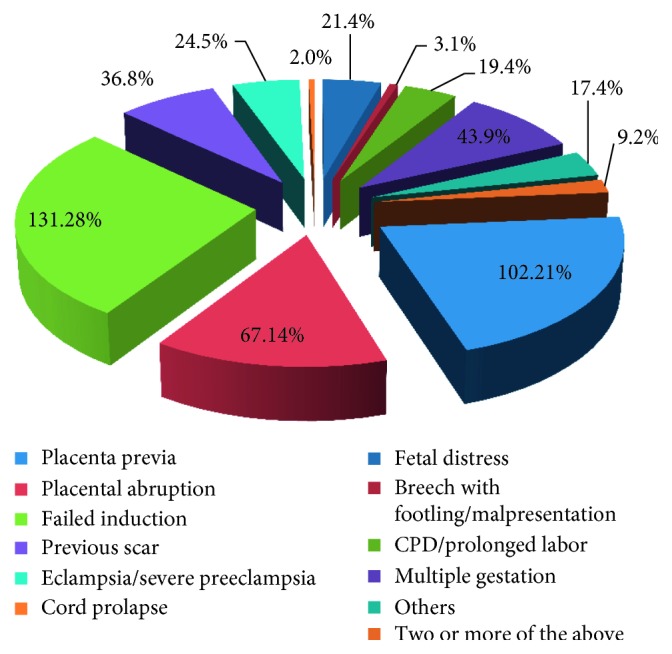
Indications for cesarean section at selected health institutions, July 2015–June 2016 (*n*=474).

Of the 474 CS deliveries, 436 (92%) were performed because of emergency conditions. But 85% of CS were due to cephalopelvic disproportion (CPD), fetal distress, malpresentation, previous CS scar, multiple gestation, and antipartum hemorrhage; 131 (28%), 102 (21%), 67 (14%), 43 (9%), 36 (8%), and 25 (5%), respectively (Figure 2).

**Figure 3:**
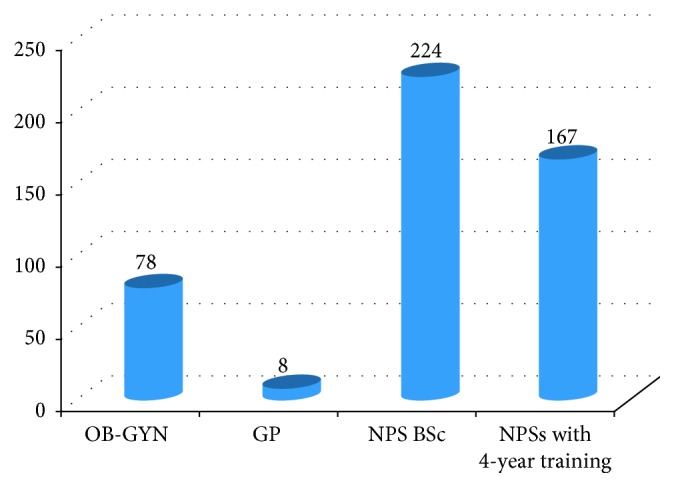
Types of staff members that performed the cesarean section in two hospitals, July 2015–June 2016 (*n*=474).

Often, spinal anesthesia and lower uterine segment transverse CS were used in 379 (79.1%) and 464 (96.9%) mothers, respectively. The majority (82%) of procedures were performed by nonphysician surgeons. The average time of the procedure was 51 (SD 18.62) minutes (Figure 3).

For 176 (37%) mothers, labor was managed by partograph, but 144 (82%) of them were completely filled. The average length of hospitalization was 5 (SD ± 2) days. Estimated average blood loss is 495 ml (SD ± 156). Of the total mothers undergone CS, only 33 (6.9%) mothers were blood transfused; 31 mothers transfusion were prescribed by NPSs.

#### 3.1.4. Complication and Death

This study revealed 40 (8.4%) deaths and 33 (6.7%) complications, totally 73 (15.2%) bad immediate CS outcomes (Table 1).

#### 3.1.5. Factors Determining Outcomes of CS Delivery

Other than the type of staff, time of hospitalization (ARR: 2.96, 95% CI (1.4, 6.26)), condition of the fetus during admission (ARR: 3.53 95% CI (1.97, 6.31)), and the type of anesthesia used for surgery (ARR: 4.19, 95% CI (2.26, 7.77)) significantly determined immediate CS birth outcomes (Table 2).

### 3.2. Discussion

This study reveals that the rate of CS, the rate of bad CS birth outcome, major indications for CS, the effect of surgical task shifting, and factors determine CS birth outcome. Incompleteness of maternal data registration imposes gap in the study, but strong association between factors and CS strengthens our findings.

The rate of CS delivery (24.5%) obtained in this study is greater than the normal range set by WHO, which is between 5 and 15% and rates reported nationally and abroad [10, 12, 20–23]. Similar to different findings, high emergency conditions (92%) are the major reasons for CS delivery [13, 15, 22]. This shows that surgical task shifting is playing a great role in Ethiopia to reduce maternal and fetal mortality because the commonest emergency conditions, such as CPD, fetal distress, malpresentation, and antipartum hemorrhage, similar to findings from different countries [12, 13, 15, 17, 18], can lead to fetal or maternal mortality if they are not managed through surgical interventions. However, in order to reduce risk of surgery, the increasing rate of CS delivery needs deep investigation.

In Ethiopia and other countries where both surgeons and NPSs are active, NPSs are performing majority (82%) of CS procedures, [17, 19, 24–26]. This means NPSs contribute in saving the lives of mothers and fetuses during emergency conditions. Moreover, they make CS service more accessible to people in need. However, out of 73 (15%) bad CS birth outcomes, 63 (13%) of them were due to NPSs. But there is no statistically significant birth outcome difference between those performed by physicians and NPSs (AOR 1.24 (95% CI: 0.55, 2.78)). This is also similar to the finding from another part of Ethiopia [12]. But there are factors affecting CS birth outcome of children, such as using general anesthesia four times likely leads to bad newborn outcome than using spinal anesthesia (AOR 4.19, 95% CI: 2.26, 7.77); this is similar with findings from Tigray, Ethiopia [12], being hospitalized for four or more days is at risk to develop bad CS birth outcome (AOR 2.96, 95% CI: 1.40, 6.26), and moreover,fetus under the distress condition during admission was more at risk for bad CS outcome than fetus under well condition during admission (AOR 3.53, 95% CI: 1.97, 6.31); this finding is similar to a study finding from Malawi [14].

## 4. Conclusion

NPSs are dominant hand for saving the life of children of Ethiopia. Using spinal anesthesia and short-time hospitalization will improve CS birth outcomes.

## Figures and Tables

**Table 1:** Immediate newborn outcome of CS delivery from July 2015 up to June 2016.

Factor	Number (%)		
Outcome of newborn	Normal live birth	401	83.7
	Live birth with distress	32	6.7
	Dead	24	5.0
	One or more death for twin	16	3.3

Death	Stillbirth	15	68.2
	Early neonatal death	7	31.8

Primary cause of stillbirth	Asphyxia and trauma	14	70
	Infection or pneumonia	1	5
	Trauma	1	5
	Other	2	10
	Unknown	1	5
	No information	1	5

**Table 2:** Factors affecting newborn outcome of cesarean delivery.

Factor		Death/distress of newborn		COR (95% CI)	AOR (95% CI)
		No	Yes		
Age	15–25	238	37	1	
	26–35	148	31	1.3 (0.8, 2.26)	
	> = 36	15	5	2.1 (0.7, 6.2)	

Residence	Urban	157	14	1	
	Rural	231	56	2.9 (1.5, 5.5)	

Parity	Nulliparous	163	24	1	
	Primiparous	103	13	0.8 (0.4, 1.7)	
	Multiparous	88	19	1.46 (0.76, 2.8)	
	Grand multiparous	47	17	2.31 (1.13, 4.7)	

Mother referred	No	212	25	1	
	Yes	173	47	2.4 (1.4, 4)	

Condition of fetus	Well	289	26	1	1
	Any sign of complication	110	47	4.9 (2.9, 8.4)	3.53 (1.97, 6.31)

Condition of mother	Stable vital sign	273	42	1	
	Critical	124	31	1.7 (0.99, 2.8)	

Time on labour	Normal	239	34	1	
	Prolonged	114	33	2 (1.2, 3.4)	

Type of anesthesia	Spinal	338	37	1	1
	General	63	36	5.2 (3, 8.9)	4.19 (2.26, 7.77)

CS classified	Emergency	365	71	1	
	Elective	27	1	5.1 (0.69, 38.7)	

Duration of procedure	≤30 minute	80	18	1	
	≥30 minute	321	55	0.68 (0.37, 1.23)	

Hospitalized date	≤3	94	12	1	1
	≥4	287	57	3.9 (2.2, 6.9)	2.96 (1.40, 6.26)

Staff	Physician	75	10	1	1
	Nonphysician	325	63	1.4 (0.7, 2.9)	1.24 (0.55, 2.78)

## Data Availability

The data used to support the findings of this study are available from the corresponding author upon request.
